# Supplementary material for: Isolation, Characterization, and Application of Clostridium sporogenes F39 to Degrade Zearalenone under Anaerobic Conditions
Source: Foods. 2022 Apr 20;11(9):1194. doi: 10.3390/foods11091194 (PMC9103434; doi:10.3390/foods11091194)
Supplement: Supplementary file 1 [file foods-11-01194-s001.zip › foods-1654857-supplementary.pdf]

Article

Isolation, Characterization, and Application of *Clostridium sporogenes* F39 to Degrade Zearalenone under Anaerobic Conditions

Supporting Documents

Congning Zhai [1,2], Yangguang Yu [1,2], Jun Han [2], Junqiang Hu [2], Dan He [2], Hongyin Zhang [1], Jianrong Shi [1,2], Sherif Ramzy Mohamed [3], Dawood H. Dawood [4], Gang Wang [2,*], and Jianhong Xu [1,*]

[1] School of Food and Biological Engineering, Jiangsu University, Zhenjiang, Jiangsu, P. R. China; 1169184176@qq.com (C.Z.); yuyangguang1997@163.com (Y.Y.); zhanghongyin126@126.com (H.Z.); shiji@jaas.ac.cn (J.S.); wanggang2015@jaas.ac.cn (G.W.); xujianhongnj@126.com (J.X.)

[2] Jiangsu Key Laboratory for Food Quality and Safety-State Key Laboratory Cultivation Base, Ministry of Science and Technology/Key Laboratory for Agro-product Safety Risk Evaluation (Nanjing), Ministry of Agriculture and Rural Affairs/Key Laboratory for Control Technology and Standard for Agro-product Safety and Quality ,Ministry of Agriculture and Rural Affairs /Collaborative Innovation Center for Modern Grain Circulation and Safety/Institute of Food Safety and Nutrition, Jiangsu Academy of Agricultural Sciences, Nanjing, Jiangsu, P. R. China; 15150537050@163.com (J.Han.); 2021216027@stu.njau.edu.cn (J.Hu.); danhe58@163.com (D.H.)

[3] Food Industries and Nutrition Research Institute, Food Toxicology and Contaminants Department, National Research Centre, Tahreer St., Dokki, Giza, 12411, Egypt; sheriframzy4@gmail.com

[4] Department of Agriculture Chemistry, Faculty of Agriculture, Mansoura University, Mansoura, 35516, Egypt; dhosni1978@yahoo.com

* Correspondence: wanggang2015@jaas.ac.cn (G.W.); xujianhongnj@126.com (J.X.)

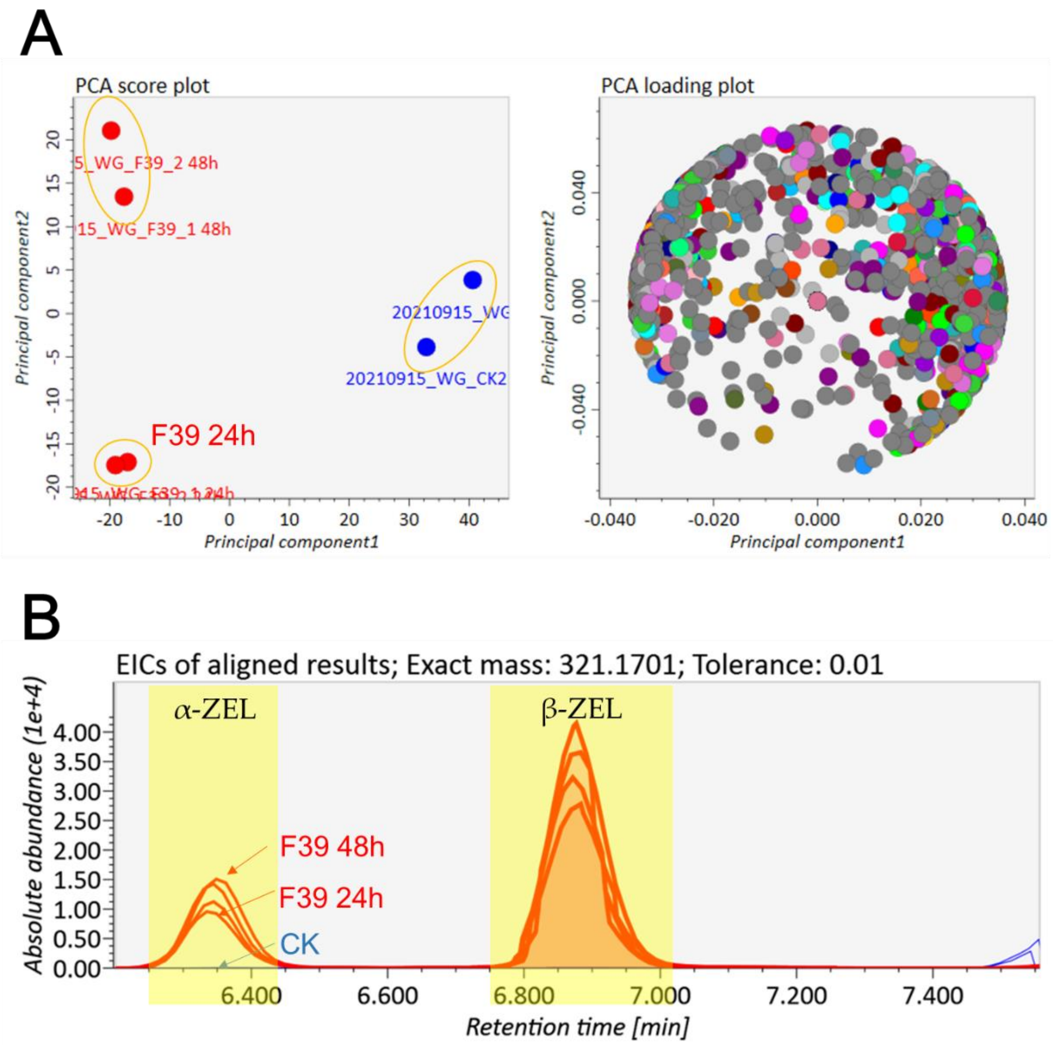

Figure S1. The LC-TOF-MS profiles of ZEN degradation product by F39. (**A**) PCA analysis of the ZEN-supplemented GAM culture with or without F39 inoculation, and (**B**) the EIC spectrum of α- and β-ZEL in the cultures of F 39.
